# Performance of Harmonic devices in surgical oncology: an umbrella review of the evidence

**DOI:** 10.1186/s12957-017-1298-x

**Published:** 2018-01-04

**Authors:** Hang Cheng, Jeffrey W. Clymer, Behnam Sadeghirad, Nicole C. Ferko, Chris G. Cameron, Joseph F. Amaral

**Affiliations:** 1 Ethicon Inc, 4545 Creek Rd, Cincinnati, OH 45242 USA; 2 Cornerstone Research Group, 3228 South Service Road, Suite 204, Burlington, Ontario L7N 3H8 Canada

**Keywords:** Harmonic scalpel, Surgical oncology, Systematic review, Meta-analysis, GRADE, AMSTAR

## Abstract

**Background:**

We performed an umbrella review of systematic reviews summarizing the evidence on the Harmonic scalpel (HS) compared with conventional techniques in surgical oncology (including lymph node dissection).

**Methods:**

We searched MEDLINE, EMBASE, and the Cochrane Database of Systematic Reviews from inception to end of March of 2017 for meta-analyses or systematic reviews of randomized trials comparing HS to conventional techniques in surgical oncology. We assessed the quality of included systematic reviews with AMSTAR (A MeaSurement Tool to Assess systematic Reviews) and assessed the certainty in evidence for each pooled outcome using GRADE (Grading of Recommendations Assessment, Development, and Evaluation).

**Results:**

We identified ten systematic reviews on breast cancer (*n* = 3), gastric cancers (*n* = 3), oral, head, and neck cancers (*n* = 1), and colon cancers (*n* = 3). Most reviews received a higher rating using AMSTAR. For operative time, systematic reviews reported a reduction of 25 to 29 min for HS compared with conventional methods across oncology types, with the exception of breast cancer where little differences were observed (very low to moderate quality of evidence (GRADE)). For blood loss and drainage volume, the majority of reviews reported statistically significant reductions with HS, and reductions ranged from 42 to 141 mL, and from 42 to 292 mL, respectively (very low to moderate quality of evidence). Hospitalization days were reported to decrease with use of HS by 0.2 to 3.2 days; however, reductions were only statistically significant for half of the included reviews (low to moderate quality of evidence). Regarding perioperative complications, two of six reviews reported a significantly reduced risk with HS use (breast cancer surgery) (moderate to high quality evidence)).

**Conclusion:**

Across surgical oncology types, the majority of included systematic reviews showed a statistically significant or numerical improvement in surgical outcomes with use of the HS compared with conventional methods. Well-designed randomized studies with large sample sizes will help to provide more precise estimates and reduce the risk of heterogeneity.

**Electronic supplementary material:**

The online version of this article (10.1186/s12957-017-1298-x) contains supplementary material, which is available to authorized users.

## Background

Various new surgical devices and technologies have been introduced in the past three decades to improve the efficiency of surgical procedures—either to achieve a range of desired tissue dissection, transection, and hemostasis or to reduce operative time and postoperative complications [1–4]. One such technological advancement included the introduction of ultrasonic cutting and coagulation of soft tissues [5, 6].

The Harmonic scalpel (HS) is a device that uses ultrasonic energy for cutting of the tissues, tissue dissection, and coagulation [6]. Numerous studies have described the advantages of ultrasonic sealing and cutting devices over conventional electrosurgery including better hemostasis with minimal thermal damage, reduced risk of nerve damage, fewer instrument changes due to the combined vessel-sealing, tissue cutting and dissecting functionality, and lower visual obstruction from mist or smoke [3, 4, 7, 8]. Hemostasis is achieved by coaptation of the vessels and sealing with a denatured protein coagulum as well as mechanically breaking tertiary hydrogen bounds in protein molecules by transducing the mechanical energy to tissue. The HS works at lower temperatures than other electrosurgical devices and has been shown to be safe with less lateral thermal damage in laparoscopic surgery [9, 10]. Many systematic reviews (SRs) and meta-analyses have demonstrated the significant clinical benefits of using HS in various types of surgeries, including a reduction in operative time, intra-operative blood loss, post-operative drainage volume, post-operation complications, and duration of hospital stay [11–17].

Systematic reviews of randomized controlled trials (RCTs) are the most rigorous source of evidence to inform clinical decision-making; however, narrow objectives of single SRs, which may only focus on certain populations or interventions, often do not allow comprehensive assessments [18, 19]. An umbrella systematic review, otherwise termed an overview of SRs, is a methodology suggested as the logical and appropriate next step when there are numerous SRs and meta-analyses on a topic. This type of review only considers the highest level of evidence, namely other SRs and meta-analyses. This allows the comparison of findings of numerous SRs and their methodological quality, to help clinicians and healthcare providers make more accurate informed decisions [18].

Surgical oncology, one of the earliest forms of cancer treatment, has expanded from that of purely therapeutic to include both palliation and prophylaxis, with new discoveries continually expanding complexities of this specialty. These surgeries usually involve the resection of diseased tissue with a suitable margin and the removal of regional lymph nodes [20]. Harmonic devices are often used in these processes for cutting, coagulation, and dissection and have been studied in randomized trials for various oncological surgeries. Several SRs and meta-analyses have been published on the outcomes associated with using Harmonic devices in surgical cancer patients such as mastectomy [13, 21, 22] and gastrectomy [12, 23, 24]. However, to our knowledge, there has been no effort to summarize the totality of the evidence, and associated limitations, across published meta-analyses. Therefore, we conducted an umbrella review of existing SRs and meta-analyses of RCTs to summarize the evidence on the performance of Harmonic devices in oncologic surgeries, assess the methodological quality, and evaluate the strength of the evidence.

## Methods

We followed the Cochrane Collaboration guideline in conducting and reporting the results of this review [25]. An a priori protocol for this study was not published but was developed for internal use. No substantive changes were made to the study design after inception. In all steps of the review, disagreements between reviewers were resolved through discussion, and if needed, by third party adjudication.

### Search strategy

We systematically searched MEDLINE, EMBASE, and Cochrane Database of Systematic Reviews from inception to the end of March of 2017 for meta-analyses or systematic reviews of RCTs. Reference lists from eligible systematic reviews and related reviews were scanned for additional citations. All searches were limited to English-language articles in humans. To assess the up-to-datedness of published systematic reviews, we searched MEDLINE, EMBASE, and Cochrane Central Register of Controlled Trials (CENTRAL) for English language RCTs published in the last 10 years based on eligibility criteria provided by included SRs. We also scanned the reference list of all relevant reviews for additional eligible RCTs. The overall search strategy is provided in Appendix 1.

### Eligibility criteria and data extraction

Two independent reviewers (BS and NF) assessed the eligibility of retrieved citations from the search strategy including articles if (i) they were SRs of RCTs (with or without meta-analysis) with clear inclusion/exclusion criteria and an explicit search strategy, (ii) assessed the effect of using a Harmonic device compared to conventional surgical techniques (e.g., monopolar electrosurgery, clamp-cut-tie, clips) in cancer patients undergoing oncologic surgeries, and (iii) reported summary measures for any of the following outcomes: operating time (min), intra-operative blood loss (mL), drainage volume (mL), duration of hospitalization (days), perioperative complications, or development of seroma.

Narrative and other types of non-systematic reviews (e.g., critical reviews, overviews, state-of-the-art reviews), clinical practice guidelines, evidence summaries, critically appraised topics, clinical paths, consumer information sheets, best practice information sheets, technical reports, and other evidence-based pieces were excluded from the review.

From each eligible study, one investigator (BS) abstracted information which was independently checked by a second investigator (NF). We extracted data on first author, year of publication, number of included RCTs and number of participants, participant diagnosis, surgical procedure, main results (effect size with 95% confidence intervals [95% CIs]), measures of heterogeneity, the risk of bias in individual RCTs, and publication bias. The rate of seroma only applied to reviews of breast cancer surgeries and was extracted separately from perioperative complications because it is a common complication and reviews consistently reported these outcomes separately.

The eligibility of retrieved RCTs was also assessed based on criteria provided in the original systematic review. In brief, RCTs were included if patients were diagnosed with cancer and randomized to the Harmonic device or conventional technique and reported information on any of the outcomes mentioned above. Two independent investigators (BS and NF) extracted data from eligible RCTs on first author, year of publication, information on intervention and comparison(s) and number of participants in each trial arm, participant diagnosis, and information on outcomes reported as primary or secondary in published SRs.

### Assessing methodological quality and certainty in evidence

The methodological quality and risk of bias of included reviews were assessed using AMSTAR (A MeaSurement Tool to Assess systematic Reviews) [26]. We also assessed the certainty in evidence for each pooled outcome from the included meta-analyses using GRADE (Grading of Recommendations Assessment, Development, and Evaluation) methodology [27]. In this approach, the certainty in evidence was categorized as high, moderate, low, or very low based on limitations in risk of bias, precision, consistency, directness, and publication bias. One investigator (BS) performed a quality assessment, which was independently checked by a second investigator (NF).

## Results

### Characteristics of included reviews and overall results

**Fig. 1:**
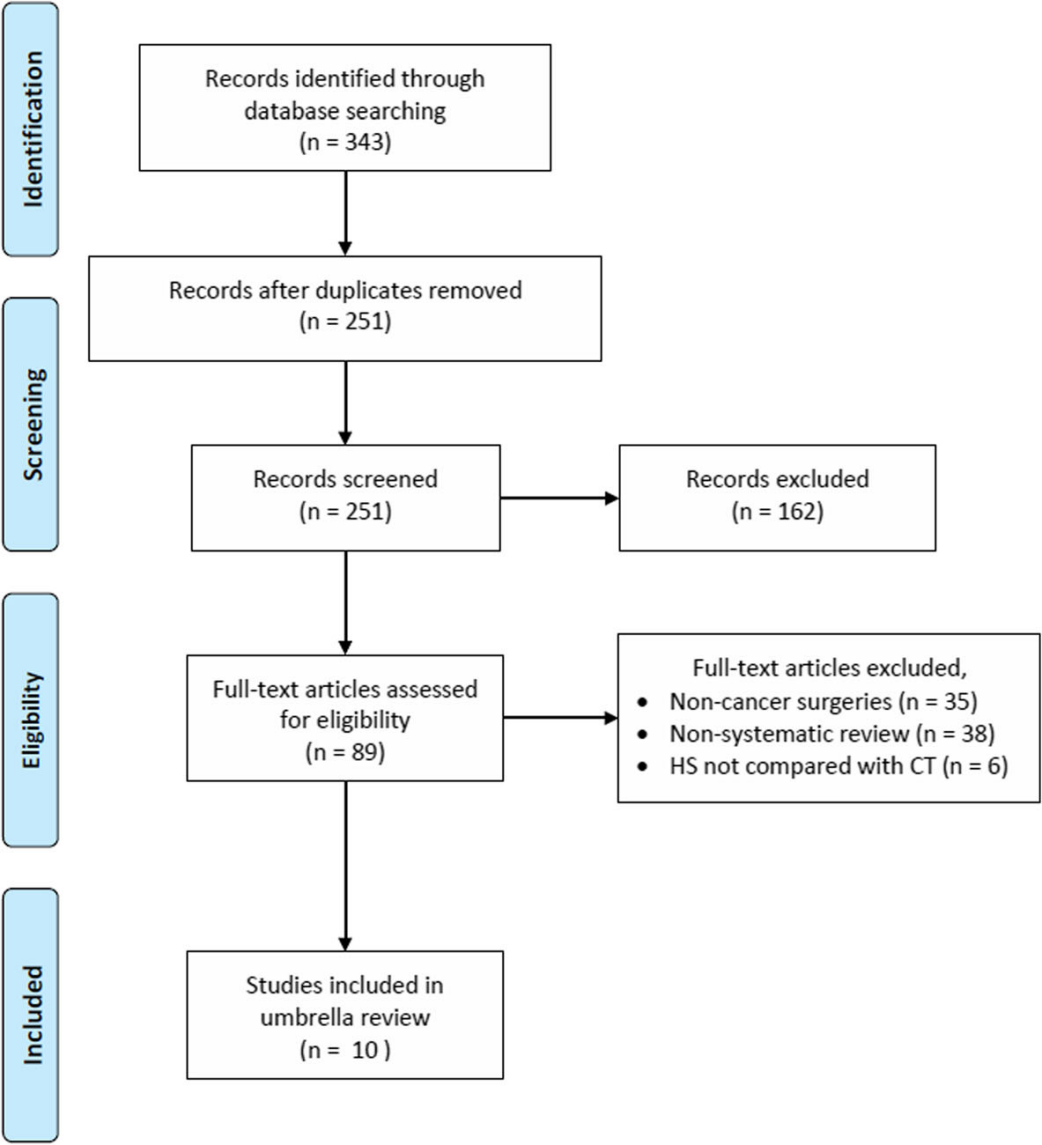
PRISMA flow chart of study selection

Excluding duplicates, a total of 89 full-text articles were screened for inclusion. Of those, ten SRs were included in this umbrella review. Figure 1 shows the process of study selection and reasons for exclusions. The SRs were published between 2011 to 2016 and included participants diagnosed with breast cancer (3 SRs) [13, 21, 22], gastric cancers (3 SRs) [12, 23, 24], oral, head, and neck cancers (1 SR) [14], and colon cancers (3 SRs) [28–30]. None of the included SRs assessed quality of evidence (certainty in evidence) using GRADE. The risk of bias was assessed in five SRs using the Cochrane risk of bias assessment tool, two SRs did not assess the risk of bias of their included RCTs, and the remaining SRs used the Jadad scale to assess the quality of included RCTs. Table 1 depicts the main characteristics of the included SRs. After accounting for overlapping trials and considering only those focusing on HS versus conventional methods, we identified 32 unique RCTs: 10 in gastric cancer; 12 in breast cancer; 7 in oral, head, and neck cancer; and 3 in colon cancer.

**Table 1:** Characteristics of included systematic reviews

Review (year)	Search period	Intervention	Comparisons	Surgical procedure	Outcomes	No. of RCTs	Methodological quality of RCTs†
Population: gastric cancer patients							
Sun (2015) [24]	June 2014	Ultrasonic dissection	Conventional electrocautery	Gastrectomy with D1/D2 LND	OR time; blood loss; post-op abdominal drainage; morbidity and mortality; post-op hospital stay; total cost	5	Low RoB
Cheng (2015) [12]	September 2013	Harmonic surgical devices	Conventional techniques		OR time; blood loss; post-op drainage volume; post-op hospital stay; blood transfusion; post-op complications	10	Moderate to low RoB
Chen (2014) [23]	September 2012	Ultrasonic scalpel	Conventional techniques		OR time; post-op complications; blood loss; abdominal drainage; post-op hospital stay; blood transfusion; GI function recovery days; no. dissected lymph nodes	7	Low quality‡
Population: breast cancer patients							
Huang (2015) [13]	June 2015	Harmonic scalpel	Electrocautery dissection	Modified radical mastectomy	Post-op drainage; seroma formation; blood loss; OR time; wound complications	7*	Low to moderate quality‡
Cheng (2016) [21]	January 1998 to May 2014	Harmonic technology	Conventional techniques	Mastectomy and BCS with LND	OR time; blood loss; chest wall drainage; post-op hospital stay; total complications; seroma and hematoma formation; wound infection; necrosis; ecchymosis	12	Moderate to low RoB
Currie (2012) [22]	2011	Ultrasonic dissection	Electrocautery dissection	Mastectomy ± LND	Total post-op drainage; seroma formation; blood loss; OR time; wound complications	6	Low to moderate quality‡
Population: oral, head, and neck cancer patients							
Ren (2015) [14]	2014	Harmonic scalpel	Conventional hemostasis	Neck dissection with LND	OR time; blood loss; post-op drainage; hospital stay	7	Moderate to low RoB
Population: colon cancer patients							
Allaix (2016) [28]	January 1999 to January 2016	Energy sources	Conventional electrosurgery	Laparoscopic colorectal resection	Quantitative analysis not performed	4**	Not assessed
Di Lorenzo (2012) [29]	1990 to June 2011	Ultrasonic energy	Radiofrequency		Quantitative analysis for comparison of Harmonic devices vs. conventional techniques not performed	5***	Not assessed
Tou (2011) [30]	March 2010	Energy sources	Conventional electrosurgery		OR time; blood loss; complications; conversion to open surgery; post-op hospital stay; total cost	6	Low RoB

*RoB* risk of bias, *OR time* operative time, *Post-op* post-operative, *LND* lymph node dissection (lymphadenectomy), *BCS* breast-conserving surgery

†Methodological quality of included RCTs based on Cochrane risk of bias assessment tool according to the information provided by published SRs

‡Used Jadad scale and/or Newcastle-Ottawa Scale for quality assessment of included RCTs

*Included four prospective comparative studies in addition to the seven RCTs

**Included three cohort studies in addition to four RCTs. Note that of the four RCTs, three compared HS and conventional

***Included two prospective and three retrospective comparative studies in addition to five RCTs. Note that of the five RCTs, three compared HS and conventional

**Fig. 2:**
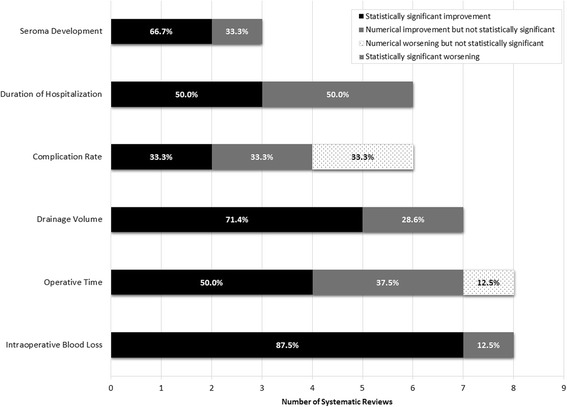
Summary of the statistical significance of systematic review results and direction of the effect. Solid bars denote improvement in outcome with Harmonic scalpel (HS) compared with conventional technique (CT). Dotted bars denote worsening of outcomes for HS compared with CT

Figure 2 provides an overview of the performance of HS compared with conventional techniques by outcome measure across surgical oncology types. In summary, most studies showed either a statistically significant or numerical improvement in outcomes with HS versus conventional methods. Results were predominantly statistically significantly in favor of the Harmonic scalpel for the outcomes of blood loss (across cancer types), drainage volume (across cancer types), and seroma development (breast cancer). For operating time and hospitalization, half of the reviews showed statistically significant improvements with HS. None of the reviews reported that HS statistically significantly worsened any outcomes.

### Gastric cancer surgeries

**Fig. 3:**
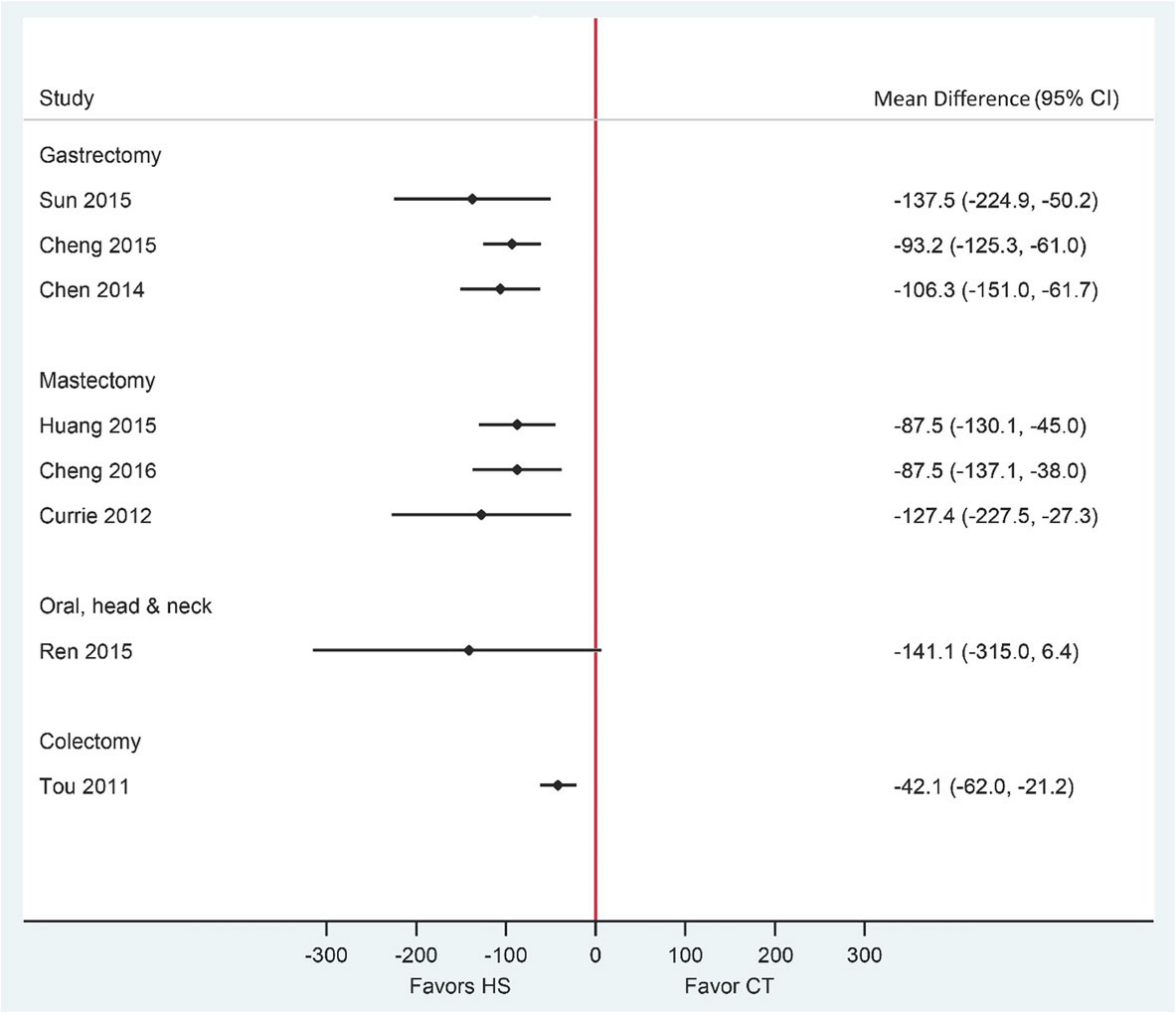
Forest plot showing the mean difference in intraoperative blood loss (mL) from published systematic reviews. Harmonic scalpel (HS) is being compared to conventional technique (CT). Horizontal bars denote 95% confidence intervals (95% CI). The solid vertical line is the line of no effect

**Fig. 4:**
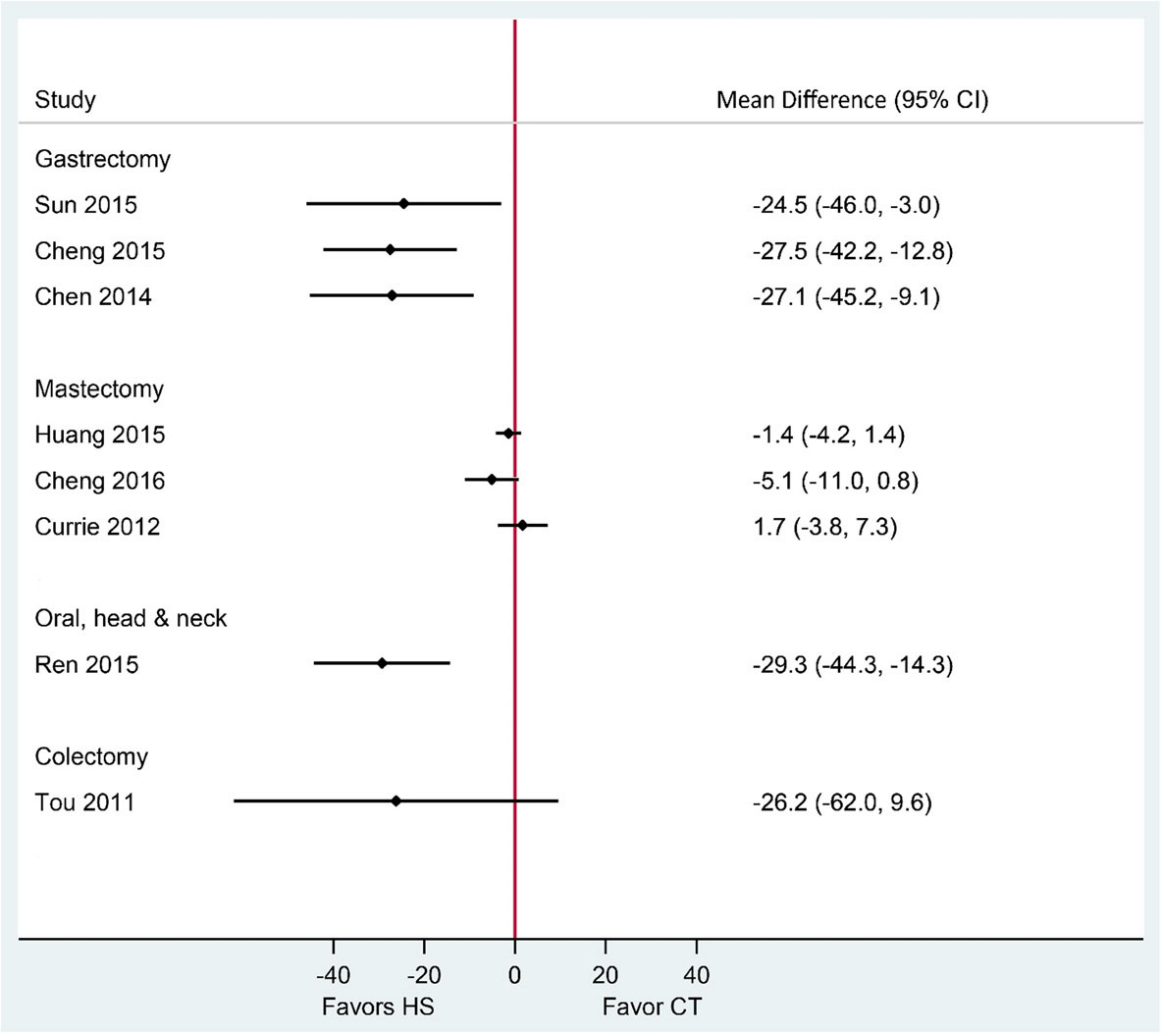
Forest plot showing the mean difference in operative time (min) from published systematic reviews. Harmonic scalpel (HS) is being compared to conventional technique (CT). Horizontal bars denote 95% confidence intervals (95% CI). The solid vertical line is the line of no effect

The quality of included SRs in this category assessed by the AMSTAR tool appeared of higher quality, with all reviews receiving a score of 7 or greater out of 11 items (Table 2). Gastrectomy with D1/D2 lymph node dissection was performed in five RCTs, and radical distal gastrectomy with lymph node dissection was performed in the other five RCTs. All included SRs in this category reported a statistically significant reduction in operative time of 24.5 to 27.5 min and statistically significant reduction in intraoperative blood loss of 93.2 to 137.5 mL using HS compared to conventional techniques (Table 3, Figs. 3 and 4). Estimates were considerably heterogeneous across published SRs. The quality of evidence (GRADE) for these outcomes varied from very low to moderate (Table 3).

**Table 2:** Methodological quality assessment of the included systematic reviews using the AMSTAR tool

Review (year)	1	2	3	4	5	6	7	8	9	10	11	Rating
Population: gastric cancer patients												
Sun (2015) [24]	No	Yes	Yes	No	No	Yes	Yes	Yes	Yes	Yes	Yes	8
Cheng (2015) [12]	No	Yes	Yes	Yes	No	Yes	Yes	Yes	Yes	Yes	Yes	9
Chen (2014) [23]	No	Yes	Yes	No	No	Yes	Yes	No	Yes	Yes	Yes	7
Population: breast cancer patients												
Huang (2015) [13]	No	CA	Yes	No	No	Yes	Yes	Yes	Yes	Yes	Yes	7
Cheng (2016) [21]	No	Yes	Yes	Yes	No	Yes	Yes	Yes	Yes	No	Yes	8
Currie (2012) [22]	No	CA	Yes	Yes	No	Yes	Yes	Yes	Yes	No	Yes	7
Population: oral, head, and neck cancer patients												
Ren (2015) [14]	No	No	Yes	No	No	Yes	Yes	Yes	Yes	Yes	Yes	7
Population: colon cancer patients												
Allaix (2016) [28]	No	Yes	No	No	No	Yes	No	NA	NA	No	Yes	3
Di Lorenzo (2012) [29]	No	Yes	No	No	No	Yes	No	NA	Yes	No	Yes	4
Tou (2011) [30]	Yes	Yes	Yes	Yes	Yes	Yes	Yes	Yes	Yes	No	Yes	10

All 11-items were scored as “Yes,” “No,” “Can’t Answer” (CA), or “Not Applicable” (NA). AMSTAR comprises the following items:

1. “A priori” design provided;

2. Duplicate study selection/data extraction;

3. Comprehensive literature search;

4. Status of publication as inclusion criteria (i.e., gray or unpublished literature);

5. List of studies included/excluded provided;

6. Characteristics of included studies documented;

7. Scientific quality assessed and documented;

8. Appropriate formulation of conclusions (based on methodological rigor and scientific quality of the studies);

9. Appropriate methods of combining studies (homogeneity test, effect model used and sensitivity analysis);

10. Assessment of publication bias (graphic and/or statistical test); and

11. Conflict of interest statement

**Table 3:** Overview of the results of included reviews comparing HS use to CT in oncologic surgeries

Review (year)	Population	Effect size (95% CI)	*P* value for difference	No. of participant (HS/CT)	Heterogeneity (*I*^2^)	Publication bias††	GRADE Certainty in evidence‡
Operative time (min)							
Sun (2015) [24]	Gastric cancer	MD − 24.5 (− 46.0 to − 3.0)	0.026	199/198	95%	Asymmetric funnel plot	Low^1, 2, 3^
Cheng (2015) [12]		MD − 27.5 (− 42.2 to − 12.8)	< 0.001	399/382	91%	Symmetric funnel plot	Moderate^1^
Chen (2014) [23]		MD − 27.1 (−45.2 to − 9.1)	0.003	172/168	91%	Symmetric funnel plot	Very low^1, 2, 4^
Huang (2015) [13]	Breast cancer	MD − 1.4 (− 4.2 to 1.4) †	0.85	333/327	74%	NS Egger’s and Begg’s tests	Very low^1, 2, 5^
Cheng (2016) [21]		MD − 5.1 (− 11.0 to 0.8)	0.09	390/391	83%	Not assessed	Moderate^1, 6^
Currie (2012) [22]		MD 1.7 (− 3.8 to 7.3) †	0.81	125/120	42%	Not assessed	Low^1, 2^
Ren (2015)[14]	Oral, head, and neck cancer	MD − 29.3 (− 44.3 to − 4.3)	< 0.001	201/205	92%	Symmetric funnel plot	Moderate^1, 7^
Tou (2011) [30]	Colon cancer	MD − 26.2 (− 62.0 to 9.6) *	0.15	94/92	87%	Not assessed	Low^1, 2, 8^
Intraoperative blood loss (mL)							
Sun (2015) [24]	Gastric cancer	MD − 137.5 (− 224.9 to − 50.2)	0.002	195/196	91%	Asymmetric funnel plot	Low^1, 2, 3^
Cheng (2015) [12]		MD − 93.2 (− 125.3 to − 61.0)	< 0.001	349/336	86%	Symmetric funnel plot	Moderate^1^
Chen (2014) [23]		MD − 106.3 (− 151.0 to − 61.7)	< 0.001	172/168	93%	Symmetric funnel plot	Very low^1, 2, 4^
Huang (2015) [13]	Breast cancer	MD − 87.5 (− 130.1 to − 45.0)†	< 0.001	226/237	92%	NS Egger’s and Begg’s tests	Low^1, 5^
Cheng (2016) [21]		MD − 87.5 (− 137.1 to − 38.0)	< 0.001	323/321	99%	Not assessed	Moderate^1^
Currie (2012) [22]		MD − 127.4 (− 227.5 to − 27.3)†	0.013	126/137	91%	Not assessed	Very Low^1, 2, 9^
Ren (2015) [14]	Oral, head, and neck cancer	MD − 141.1 (− 315.0 to 6.4)	0.112	153/151	100%	Symmetric funnel plot	Moderate ^2, 10^
Tou (2011) [30]	Colon cancer	MD − 42.1 (− 62.0 to − 21.2)	< 0.001	94/92	0.0%	Not assessed	Moderate^2^
Drainage volume (mL)							
Sun (2015) [24]	Gastric cancer	MD − 292.3 (− 708.3 to 123.7)	0.168	148/145	77%	Asymmetric funnel plot	Low^1, 2, 3^
Cheng (2015) [12]		MD − 138.8 (− 177.6 to − 100.1)	< 0.001	375/359	94%	Symmetric funnel plot	Moderate^1^
Chen (2014) [23]		MD − 74.6 (− 95.2 to − 54.0)	< 0.001	69/69	84%	Symmetric funnel plot	Very low^1, 2, 4^
Huang (2015) [13]	Breast cancer	MD − 211.6 (− 353.9 to − 69.2)†	0.004	258/269	91%	NS Egger’s and Begg’s tests	Moderate^1^
Cheng (2016) [21]		MD − 42.1 (− 65.9 to − 18.9)	< 0.001	127/129	87%	Not assessed	Low^1, 2^
Currie (2012) [22]		MD − 141.5 (− 335.9 to 53.0)†	0.154	138/149	81%	Not assessed	Low^1, 2^
Ren (2015) [14]	Oral, head, and neck cancer	MD − 64.9 (− 110.4 to − 19.3)	0.005	191/195	97%	Symmetric funnel plot	Low^1, 2^
Duration of hospitalization (days)							
Sun (2015) [24]	Gastric cancer	MD − 2.1 (− 4.0 to − 0.2)	0.027	50/50	0.0%	Asymmetric funnel plot	Moderate^2^
Cheng (2015) [12]		MD − 0.6 (− 2.5 to 1.2)	0.509	81/81	65%	Symmetric funnel plot	Low^1, 2^
Chen (2014) [23]		MD − 3.2 (− 6.3 to − 0.1)	0.040	20/20	–	Symmetric funnel plot	-**
Cheng (2016) [21]	Breast cancer	MD − 1.4 (− 2.4 to − 0.4)	0.007	184/186	98%	Not assessed	Low^1, 2^
Ren (2015) [14]	Oral, head, and neck cancer	MD − 0.21 (− 0.48 to 0.07)	0.142	79/81	0.0%	Symmetric funnel plot	Moderate^2^
Tou (2011) [30]	Colon cancer	MD − 0.42 (− 0.84 to 0.00)	0.051	94/92	0.0%	Not assessed	Moderate^2^
Overall perioperative complications							
Cheng (2015) [12]	Gastric cancer	RR 0.58 (0.3 to 1.0)	0.059	235/229	12.0%	Symmetric funnel plot	High
Chen (2014) [23]		RR 0.75 (0.4 to 1.3)	0.276	126/121	0.0%	Symmetric funnel plot	Moderate^2^
Huang (2015) [13]	Breast cancer	RR 0.38 (0.2 to 0.6)	0.01	199/209	23.0%	NS Egger’s and Begg’s tests	High
Cheng (2016) [21]		RR 0.5 (0.3 to 0.8)	0.002	NR	0.0%	Not assessed	Moderate^2^
Currie (2012) [22]		OR 1.6 (0.7 to 3.7)	0.3	NR	35.0%	Not assessed	Very low^1, 2, 9^
Tou (2011) [30]	Colon cancer	RR 1.28 (0.7 to 2.3)	0.395	106/103	0.0%	Not assessed	Moderate^2^
Seroma development							
Huang (2015) [13]	Breast cancer	RR 0.5 (0.3 to 0.7)	< 0.001	82/125	0.0%	NS Egger’s and Begg’s tests	Moderate^2^
Cheng (2016) [21]		RR 0.5 (0.4 to 0.7)	< 0.001	410/411	25.0%	Not assessed	High
Currie (2012) [22]		OR: 0.8 (0.4 to 1.4)	0.368	45/49	0.0%	Not assessed	Low^2, 9^

*HS* Harmonic devices, *CT* conventional techniques, *MD* mean difference, *SMD* standardized mean difference, *RR* risk ratio, *OR* odds ratio, *NR* not reported, *NS* non-significant

*For the comparison of monopolar electrocautery scissors and ultrasonic coagulating shears

**Only one study in this category

†Original SRs reported SMD. Mean differences were calculated using data provided in forest plots of published SRs

††An asymmetric funnel plot or significant Egger’s or Begg’s test indicates the possibility of publication bias

‡ GRADE Working Group grades of evidence:

High certainty: We are very confident that the true effect lies close to that of the estimate of the effect;

Moderate certainty: We are moderately confident in the effect estimate: The true effect is likely to be close to the estimate of the effect, but there is a possibility that it is substantially different;

Low certainty: Our confidence in the effect estimate is limited: The true effect may be substantially different from the estimate of the effect;

Very low certainty: We have very little confidence in the effect estimate: The true effect is likely to be substantially different from the estimate of effect

^1^ Given the substantial heterogeneity in the pooled estimate, we rated down for inconsistency

^2^ For continuous outcomes, GRADE guideline suggests downgrading for sample size less than 400

^3^ We decided not to rate down for publication bias as Cochrane suggests tests for funnel plot asymmetry should be used only when there are at least ten studies included in the meta-analysis

^4^ The quality of RCTs was assessed using Jadad scale, and their scores were located at the low level, mainly due to the absence of randomization details

^5^ The quality of RCTs was assessed using Jadad scale. We decided not to rate down as four out of seven RCTs were categorized as high quality

^6^ We decided not to rate down for risk of bias as only 1 out of 12 RCTs were considered high risk of bias

^7^ We decided not to rate down for risk of bias as four out of seven RCTs were identified as being of high or moderate quality

^8^ We decided not to rate down for risk of bias as only one out of six included RCTs were considered high risk of bias

^9^ We decided to rate down for risk of bias as four out of six included RCTs were with a high risk of bias

^10^ Although effect estimates and their 95% Cis from RCTs did not overlap, we decided not to rate down for inconsistency as all had the same direction and *I*^2^ for authors sensitivity analysis is zero

**Fig. 5:**
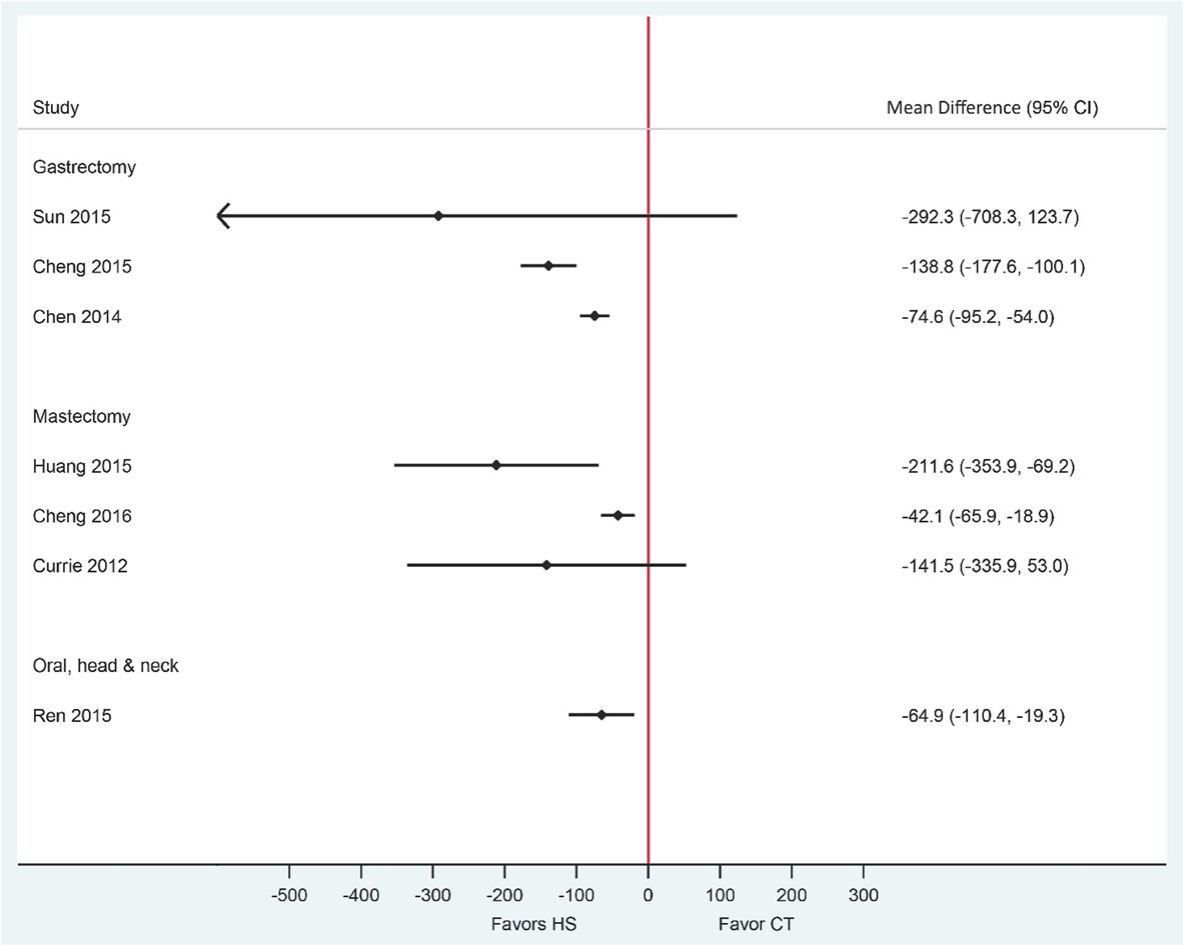
Forest plot showing the mean difference in drainage volume (mL) from published systematic reviews. Harmonic scalpel (HS) is being compared to conventional technique (CT). Horizontal bars denote 95% confidence intervals (95% CI). The solid vertical line is the line of no effect

**Fig. 6:**
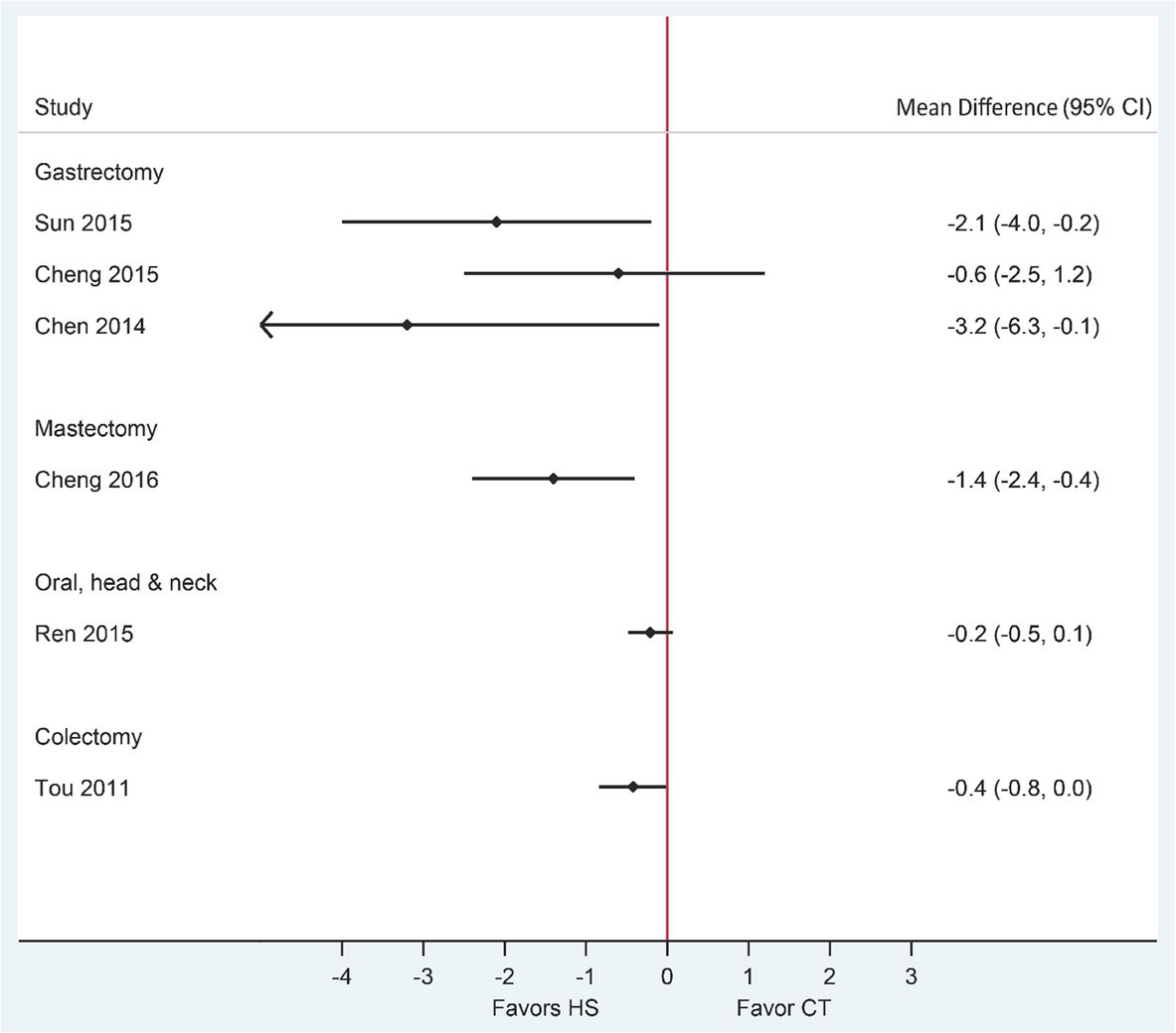
Forest plot showing the mean difference in duration of hospitalization (days) from published systematic reviews. Harmonic scalpel (HS) is being compared to conventional technique (CT). Horizontal bars denote 95% confidence intervals (95% CI). The solid vertical line is the line of no effect

**Fig. 7:**
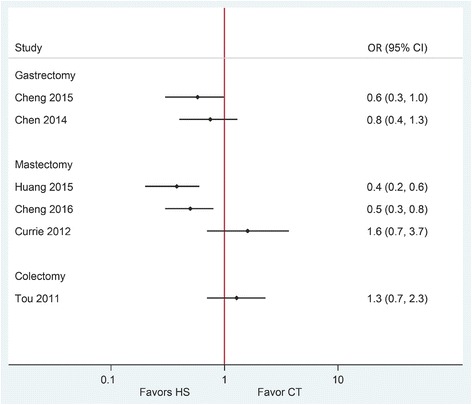
Forest plot showing the odds ratio (OR) for the complication rate from published systematic reviews. Harmonic scalpel (HS) is being compared to conventional technique (CT). Horizontal bars denote 95% confidence intervals (95% CI). The solid vertical line is the line of no effect

Drainage volume and duration of hospitalization were reported to be statistically significantly reduced in two of the three SRs when comparing HS with conventional techniques (reduction ranged from 74.6 to 292.3 mL (very low to moderate quality of evidence) and 0.6 to 3.2 days (low to moderate quality of evidence), respectively) (Figs. 5 and 6). Both SRs that reported on overall perioperative complications showed numerical risk reductions for this outcome, albeit non-statistically significant, with moderate and high certainty in evidence and low heterogeneity (Fig. 7, Table 3).

Assessing up-to-datedness of included SRs in gastric cancer surgery, we found that two new RCTs were published in 2016 [31, 32]. They reported reductions that were not statistically significant in operative time, blood loss, drainage volume, and hospitalization for patients who underwent surgeries with HS compared to conventional techniques (Additional file 1: Table S1).

### Breast cancer surgeries

The quality of three included SRs in this category assessed by the AMSTAR tool appeared of higher quality, with all reviews receiving a score of 7 or greater out of 11 items (Table 2). SRs in this category compared the use of HS with conventional techniques in radical modified mastectomy, mastectomy, and breast-conserving surgery with lymphadenectomy in breast cancer patients. The results of SRs in this population were almost always consistently in favor of the Harmonic device across outcomes, except for operative time (Table 3, Figs. 3, 4, 5, 6, and 7). Very low, low, and moderate evidence quality was reported for published SRs which showed no difference in operative time. Pooled estimates from all SRs showed a statistically significant reduction in intraoperative blood loss when HS was compared with conventional techniques (Fig. 3). Estimates were heterogeneous with the quality of evidence (GRADE) ranging from very low to moderate (Table 3).

Drainage volume, the risk of overall perioperative complications, and risk of seroma development were statistically significantly reduced with HS in two of the three SRs. Pooled estimates ranged from a reduction of 42.1 to 211.6 mL in drainage volume, with moderate and low quality of evidence (GRADE) (Fig. 5). Pooled estimates for the risk of overall perioperative complications and seroma development showed statistically significant reductions of over 50% in two SRs with high and moderate quality of evidence (Table 3, Fig. 7). Duration of hospitalization was reported only in one SR and showed a statistically significant reduction of 1.4 days when HS was compared with conventional techniques (low quality evidence) (Table 3, Fig. 6).

Assessing up-to-datedness of SRs, we found four RCTs that were not included in the published SRs. Two RCTs reported operative time, blood loss, drainage volume, and risk of seroma formation [33, 34]; only one reported duration of hospitalization [34]; and two others only reported drainage volume [35, 36]. The trials showed statistically significant improvements in favor of HS usage compared to conventional techniques for most of these surgical outcomes (Additional file 1: Table S1).

### Oral, head, and neck cancer surgeries

We found one moderate quality SR including seven RCTs that compared HS use in oral, head, and neck cancer surgeries with conventional techniques, which reported pooled estimates for four of six outcomes of interest [14]. This SR received a rating of 7 out of 11 by the AMSTAR tool. Of seven unique RCTs included in this SR, four RCTs included patients diagnosed with squamous cell carcinoma of oral cavity, oropharynx, larynx, or hypopharynx; two RCTs included patients diagnosed with thyroid papillary carcinoma; and one RCT included patients diagnosed with laryngeal carcinomas. In all RCTs, lymph node dissection (level I to IV) was part of the surgical procedure. HS use showed a statistically significant reduction in operative time and drainage volume and a numerical reduction in intraoperative blood loss and duration of hospitalization (Table 3, Figs. 3, 4, 5, and 6). Heterogeneity was observed for all outcomes except the duration of hospitalization. Specifically, the review demonstrated that HS was associated with a reduction of 29.3 min in operative time (moderate quality evidence), a reduction of 141.1 mL in blood loss (moderate quality evidence), a reduction of 64.9 mL in drainage volume (low quality of evidence), and a reduction of 0.2 days in duration of hospitalization (moderate quality evidence) **(**Table 3). Outcomes were not reported for perioperative complications.

We only found one eligible RCT that was not included in this SR. This study reported a statistically significant reduction in operative time, drainage volume, and duration of hospitalization (Additional file 1: Table S1) [37].

### Colon cancer surgeries

The quality of included SRs in this category varied considerably with two lower quality SRs [28, 29] and one higher quality Cochrane Review [30] according to AMSTAR (Table 2). Included trials in these SRs were comprised of a mixed population of patients with benign and malignant colon diseases (including diverticulitis, polyps, colorectal adenoma and adenocarcinoma, and epidermoid carcinoma). Only one of the included SRs performed a meta-analysis. Pooled estimate showed a non-statistically significant reduction of 26.2 min in operative time with low quality of evidence (Fig. 4), a statistically significant reduction of 42.1 mL in blood loss (moderate quality evidence) (Fig. 3), a non-statistically significant reduction of 0.4 days in duration of hospitalization (moderate quality evidence) (Fig. 6), and a non-statistically significant 28% increase in risk of overall perioperative complications (moderate quality evidence) (Fig. 7)(Table 3).

Assessing up-to-datedness of included SRs in this category, we found two new eligible RCTs reporting a non-statistically significant reduction in operative time in one study and a statistically significant reduction in operative time, drainage volume, and duration of hospitalization in the other (Additional file 1: Table S1) [38, 39].

## Discussion

This umbrella review examined a wide range of outcomes from ten SRs and meta-analyses on the effects of using HS compared with conventional methods in surgical oncology. To our knowledge, this is the first attempt to comprehensively summarize and perform a critical appraisal and quality assessment of available evidence related to HS use in surgical oncology. We used state-of-the-art GRADE methodology to assess the certainty of evidence for each outcome from included SRs. Despite the variations across populations, surgical procedures, and outcome assessments (i.e., certainty of evidence ranging from very low to high quality), favorable results for the HS were evident across several outcomes among the included SRs. Overall, the use of HS was associated with operative time reductions of close to 30 min across oncology types, with the exception of breast cancer where little differences were observed. For blood loss and drainage volume, most reviews reported statistically significant reductions with HS. Hospitalization days were also reported to decrease with HS use; however, results were statistically significant for only half of the included reviews. Reductions in perioperative complications, such as seroma, were most notable with HS for breast cancer surgery.

Almost all the included SRs in this umbrella review performed meta-analyses to obtain pooled estimates. Heterogeneity was observed in several of the meta-analyses (*I*^2^ > 40%), which may be due to variations in surgical procedures, methods used for measuring outcomes, and some studies with smaller sample sizes. Operating time may vary considerably depending on whether the skin flap is considered as a part of the surgery or not or if time was recorded from the first cutaneous incision. Ren et al. performed a sensitivity analysis excluding RCTs that measured operative time differently and found that the heterogeneity reduced considerably, while the effect measure remained significant [14]. For intraoperative blood loss, individual RCTs used various subjective methods for measurement, including weighing or counting used sponges, evaluating the volume of blood in the aspirator, a combination of both, or surgeons’ subjective appraisal [39–44]. The observed heterogeneity in pooled estimates of drainage volume may be partially due to the study surgeon’s decision on number of drains, various methods of recording the amount of drainage, the variation in the recording period, whether lymph node dissection was performed, and the number of removed nodes [13, 14, 45].

The reduction in operative time of oncologic surgeries with HS may be due to multiple factors. HS use provides more visibility and less smoke, making first-pass hemostasis more sufficient and the dissection more precise [6, 46]. Many vessels and lymphatics need to be cut and coagulated during oncologic surgeries. Given this, HS can decrease the operative time compared with relative time-consuming tools, like monopolar electrocautery and thread ligation, as it provides simultaneous cutting and sealing of vessels. Electrocautery has limitations in sealing vessels with > 1–2 mm in diameter [47, 48] and produces a large degree of smoke [46–48]. HS can help to overcome these shortcomings and provide a visible plane for the surgeons. The shorter operative time with HS may also partially be explained by the reduced instrument changes and avoidance of sequelae with changing instruments, which can be an important issue in complicated, long surgeries such as in oncology [49, 50]. In mastectomy, although HS provided a significant benefit in reduction of seroma and blood loss, there was not a substantial reduction in operative time, which may be due to the fact that this is a relatively short procedure, and larger sample sizes may be needed to detect any benefit. In essence, reliable and precise dissections and scaling/cutting with minimal instrument exchanges should lead to operative time savings, particularly in laparoscopic procedures where these maneuvers are more challenging.

Although not formally assessed as an outcome in this umbrella review, thermal damage is important to consider in context of hemostasis outcomes. Lateral thermal spread of energy results in damage of tissues near the target site and can occur across procedures. An important goal in both open and laparoscopic electrosurgery is to minimize thermal damage to surrounding tissues and to increase speed without compromising tissue integrity. Studies have demonstrated that ultrasonic energy delivered through a harmonic scalpel is safe and produces minimal damage to the surrounding tissue compared with some conventional methods (i.e., monopolar diathermy) [9, 10].

Most surgical oncology procedures involve partial or radical removal of regional lymph nodes at the time of resection of primary cancer [51–53]. This adds to the complexity of the surgical procedure as well as increasing the potential risk of overall perioperative complications, such as drainage volume, seroma formation, nerve injury, necrosis, and/or surgical site infection [23, 24, 53, 54]. Harmonic technology is reported to facilitate lymph node dissection, [55] while electrosurgery has been shown to damage subdermal vascular plexus, cause incomplete occlusion of vascular and lymphatic channels [56, 57], and fail to seal the lymphatics adequately [58]. The reduction in postoperative drainage volume and seroma formation comparing HS use with conventional surgical techniques may be due to the thrombosis of subdermal vessels and inadequately sealed lymphatics caused by electrosurgery [59]. Moreover, HS might close vascular and lymphatic channels more precisely by breaking hydrogen bonds to coagulate protein which might be related to reduce the inflammatory response and result in preventing seroma development, lymphatic spillage, and reduction of drainage volume [60–62]. Many of the lymph nodes are usually known to be located closely along the vessels and/or nerves, where delicate and precise operations are crucial. HS has thin jaws that can allow tissue dissection, cut off, and coagulation in a relatively narrow and deep space more efficiently. Another explanation is that HS is known to produce less tissue damage and concomitant inflammatory response [63].

We assessed the quality of included SRs with the AMSTAR tool with most demonstrating higher quality results, with scores of 7 or greater out of 11 points. One of the two high-quality SRs was a Cochrane SR, which are usually very detailed in reporting their methods and results, with publication of protocols a priori. Most of the SRs did not have an a priori design (i.e., registered or published protocol), did not search for grey literature, and did not provide the list of excluded RCTs. Also, none of the SRs assessed the quality (certainty) of evidence using GRADE methodology for outcomes. The AMSTAR tool helps the reader to assess critical components that an SR should report for appropriate interpretation of the results and their implications [26] and can be helpful in observing the variation in quality across systematic reviews within an umbrella review.

We used GRADE methodology to assess the quality (certainty) of evidence for each outcome from included SRs. Based on the available evidence, SRs can provide a (quantified) synthesis of the benefits and harms of a certain intervention; however, this may not be sufficient for making well-informed decisions [64]. Clinical decision making can be influenced not only by the effect estimates for benefits and harms but also by the certainty in these estimates. GRADE methodology helps with assessing the certainty in those estimates, providing a sensible gradient of the usefulness of an estimate of the magnitude of intervention effects [65]. GRADE uses four levels for quality of evidence (high, moderate, low, and very low) considering study limitations, the inconsistency of results, indirectness of evidence, imprecision, and reporting bias. These levels imply a gradient of certainty in estimates of treatment effect, and thus a gradient in the consequent strength of inference [64, 65]. The quality of evidence for approximately half of the outcomes from SRs included in our umbrella review was evaluated to be moderate or high. The main reasons for downgrading were the inconsistency of the results due to the observed heterogeneity and imprecision due to the low sample size of included RCTs.

### Limitations

Our umbrella review relied on results reported within the previously published SRs; as such, it is based on their findings and hence accepts the quality and accuracy of these studies at face value. It is possible that the SRs may have inappropriately combined conceptually heterogeneous studies or used inappropriate statistical methods. We tried to address these issues by assessing the quality of SRs using the AMSTAR tool and assessing the certainty in evidence using GRADE methodology. What we found though was that despite variation in surgical oncology types and grading of evidence findings, results were relatively consistent, as most reviews showed a statistically significant or numerical improvement in surgical outcomes for HS compared with conventional techniques. Furthermore, included SRs may have missed studies, although this is unlikely to have influenced our finding as we searched for potentially eligible RCTs and assessed the up-to-datedness of included SRs. Meta-analyses were not performed using the updated RCTs; however, upon review of the data, results of these RCTs generally aligned with the SRs. Overall, the main shortcoming of the included SRs was the low number of publications available in the literature (i.e., lowest for oral, head and neck as well as colon cancer) and the heterogeneous nature of these studies. Future research should focus on improving methodology of randomized trials evaluating HS in surgical oncology and increasing study sample sizes to help reduce the risk of heterogeneity. Finally, cost outcomes were not assessed in our review, although some resource use (e.g., hospital length of stay) was included. Another goal of future research could be to explore how the clinical findings of our umbrella review translate into economic results from the hospital perspective in surgical oncology.

## Conclusion

This umbrella review summarized and assessed the evidence on the use of HS compared to conventional techniques in surgical oncology. This is the first comprehensive systematic review of systematic reviews of Harmonic technology in this space. Across surgical oncology types, the majority of included systematic reviews showed a statistically significant or numerical improvement in surgical outcomes with use of the HS compared with conventional methods, and no review showed a statistically significant worsening of outcomes. Our study findings are however limited by study heterogeneity, and future randomized trials should improve upon study methodology to help minimize this limitation. We hope that the evidence from this review can help support decision-making for surgeons and hospitals in their choice of the most appropriate instruments to suit their needs in surgical oncology. Translation of clinical findings into economic implications can help to further support hospital decision-making needs.

## Appendix 1

Search strategy for Harmonic or ultrasonic studies

Database: Embase < 1988 to 2017 Week 8 >, Epub Ahead of Print, In-Process & Other Non-Indexed Citations, Ovid MEDLINE(R) Daily and Ovid MEDLINE(R) < 1946 to 2017 Week 8 >, and Cochrane library

Search Strategy:

1 (harmonic* adj2 (blade? or dissect* or hook? or incis* or scalpel? or shear? or scalpel?)).tw,kw. (2454)

2 (ultrasonic* adj2 (blade? or dissect* or hook? or incis* or scalpel? or shear? or scalpel?)).tw,kw. (2161)

3 (ultrasound* adj2 (blade? or dissect* or hook? or incis* or scalpel? or shear? or scalpel?)).tw,kw. (838)

4 ultracision?.tw,kw. (376)

5 ultra-cision?.tw,kw. (5)

6 (harmonic* adj (ACE* or Focus* or Synergy* or Wave*)).tw,kw. (812)

7 “ACE+ 7”.tw,kw. (21)

8 “Harmonic 7”.tw,kw. (3)

9 “CS 14-C”.tw,kw. (1)

10 “HD 1000i Shears”.tw,kw. (0)

11 HF005.tw,kw. (1)

12 Ultrasonic Surgical Procedures/ (654)

13 ((ultrason* or ultrasound*) adj2 surg* adj3 (device* or instrument* or procedur* or technique*)).tw,kw. (306)

14 or/1-13 (6556)

15 exp Animals/ not (exp Animals/ and Humans/) (13727370)

16 14 not 15 (4336)

17 (comment or editorial or interview or news or newspaper article).pt. (1671785)

18 (letter not (letter and randomized controlled trial)).pt. (1762745)

19 16 not (17 or 18) (4233)

20 limit 19 to systematic reviews [Limit not valid in Embase; records were retained] (1899)

21 meta analysis.pt. (73990)

22 exp meta-analysis as topic/ (49955)

23 (meta-analy* or metanaly* or metaanaly* or met analy* or integrative research or integrative review* or integrative overview* or research integration or research overview* or collaborative review*).tw,kw. (242010)

24 (systematic review* or systematic overview* or evidence-based review* or evidence-based overview* or (evidence adj3 (review* or overview*)) or meta-review* or meta-overview* or meta-synthes* or rapid review* or “review of reviews” or technology assessment* or HTA or HTAs).tw,kw. (282350)

25 exp Technology assessment, biomedical/ (20902)

26 (cochrane or health technology assessment or evidence report).jw. (34521)

27 ((indirect* or mixed or multi-treatment*) adj2 compar*).tw,kw. (8665)

28 ((network* or network-based) adj (MA or MAs)).kw,tw. (11)

29 or/21-28 (519420)

30 19 and 29 (65)

31 20 or 30 [SYSTEMATIC REVIEWS] (1908)

32 (controlled clinical trial or randomized controlled trial).pt. (519165)

33 clinical trials as topic.sh. (179761)

34 exp Randomized Controlled Trials as Topic/ (231226)

35 (randomi#ed or randomly or RCT$1 or placebo*).tw,kw. (1740069)

36 ((singl* or doubl* or trebl* or tripl*) adj (mask* or blind* or dumm*)).tw,kw. (316622)

37 trial.ti. (354659)

38 or/32-37 (2254804)

39 19 and 38 [RCTs] (498)

40 controlled clinical trial.pt. (91760)

41 Controlled Clinical Trial/ or Controlled Clinical Trials as Topic/ (540950)

42 (control* adj2 trial*).tw,kw. (436814)

43 Non-Randomized Controlled Trials as Topic/ (10101)

44 (nonrandom* or non-random* or quasi-random* or quasi-experiment*).tw,kw. (91083)

45 (nRCT or nRCTs or non-RCT$1).tw,kw. (1182)

46 Controlled Before-After Studies/ (167992)

47 (control* adj3 (“before and after” or “before after”)).tw,kw. (7222)

48 Interrupted Time Series Analysis/ (152036)

49 (time series adj3 interrupt*).tw,kw. (3539)

50 (pre- adj3 post-).tw,kw. (147710)

51 (pretest adj3 posttest).tw,kw. (7838)

52 Historically Controlled Study/ (187204)

53 (control* adj2 stud$3).tw,kw. (415123)

54 Control Groups/ (255317)

55 (control* adj2 group$1).tw,kw. (895570)

56 trial.ti. (354659)

57 or/40-56 (2531267)

58 19 and 57 [NON-RCTs] (397)

59 31 or 39 or 58 [ALL STUDY DESIGNS] (2894)

60 59 use ppez (1044) [MEDLINE RECORDS]

61 harmonic.dv. (603)

62 (harmonic* adj2 (blade? or dissect* or hook? or incis* or scalpel? or shear? or scalpel?)).tw,kw. (2454)

63 (ultrason* adj2 (blade? or dissect* or hook? or incis* or scalpel? or shear? or scalpel?)).tw,kw. (2300)

64 (ultrasound* adj2 (blade? or dissect* or hook? or incis* or scalpel? or shear? or scalpel?)).tw,kw. (838)

65 ultracision?.tw,kw. (376)

66 ultra-cision?.tw,kw. (5)

67 (harmonic* adj (ACE* or Focus* or Synergy* or Wave*)).tw,kw. (812)

68 “ACE+ 7”.tw,kw. (21)

69 “Harmonic 7”.tw,kw. (3)

70 “CS 14-C”.tw,kw. (1)

71 “HD 1000i Shears”.tw,kw. (0)

72 HF005.tw,kw. (1)

73 ultrasound surgery/ (387)

74 ((ultrason* or ultrasound*) adj2 surg* adj3 (device* or instrument* or procedur* or technique*)).tw,kw. (306)

75 or/61-74 (6828)

76 exp animal experimentation/ or exp. models animal/ or exp. animal experiment/ or nonhuman/ or exp vertebrate/ (39067187)

77 exp human/ or exp human experimentation/ or exp human experiment/ (31426764)

78 76 not 77 (7641574)

79 75 not 78 (6291)

80 editorial.pt. (906336)

81 letter.pt. not (letter.pt. and randomized controlled trial/) (1757763)

82 79 not (80 or 81) (6158)

83 meta-analysis/ (221672)

84 “systematic review”/ (139042)

85 “meta analysis (topic)”/ (34527)

86 (meta-analy* or metanaly* or metaanaly* or met analy* or integrative research or integrative review* or integrative overview* or research integration or research overview* or collaborative review*).tw,kw. (242010)

87 (systematic review* or systematic overview* or evidence-based review* or evidence-based overview* or (evidence adj3 (review* or overview*)) or meta-review* or meta-overview* or meta-synthes* or rapid review* or “review of reviews” or technology assessment* or HTA or HTAs).tw,kw. (282350)

88 biomedical technology assessment/ (19793)

89 (cochrane or health technology assessment or evidence report).jw. (34521)

90 ((indirect* or mixed or multi-treatment*) adj2 compar*).tw,kw. (8665)

91 ((network* or network-based) adj (MA or MAs)).kw,tw. (11)

92 or/83-91 (561546)

93 82 and 92 [SYSTEMATIC REVIEWS] (132)

94 randomized controlled trial/ or controlled clinical trial/ (1109541)

95 exp “clinical trial (topic)”/ (256945)

96 (randomi#ed or randomly or RCT$1 or placebo*).tw,kw. (1740069)

97 ((singl* or doubl* or trebl* or tripl*) adj (mask* or blind* or dumm*)).tw,kw. (316622)

98 trial.ti. (354659)

99 or/94-98 (2404037)

100 82 and 99 [RCTs] (821)

101 exp controlled clinical trial/ (1109665)

102 exp “controlled clinical trial (topic)”/ (125448)

103 (control* adj2 trial*).tw,kw. (436814)

104 (nonrandom* or non-random* or quasi-random* or quasi-experiment*).tw,kw. (91083)

105 (nRCT or nRCTs or non-RCT$1).tw,kw. (1182)

106 (control* adj3 (“before and after” or “before after”)).tw,kw. (7222)

107 time series analysis/ (23164)

108 (time series adj3 interrupt*).tw,kw. (3539)

109 (pre- adj3 post-).tw,kw. (147710)

110 (pretest adj3 posttest).tw,kw. (7838)

111 controlled study/ (5135971)

112 (control* adj2 stud$3).tw,kw. (415123)

113 control group/ (255317)

114 (control* adj2 group$1).tw,kw. (895570)

115 trial.ti. (354659)

116 or/101-115 (6991487)

117 82 and 116 [NON-RCTs] (1248)

118 93 or 100 or 117 [ALL STUDY DESIGNS] (2829)

119 118 use emed (1897) [EMBASE RECORDS]

120 60 or 119 [BOTH DATABASES] (2941)

121 remove duplicates from 154 (2081) [TOTAL UNIQUE RECORDS]

122 121 use ppez [MEDLINE UNIQUE RECORDS] (1016)

123 121 use emed [EMBASE UNIQUE RECORDS] (1065)

## Additional file

Additional file 1: Table S1. Overview of eligible RCTs not included in systematic reviews. (DOCX 28 kb)

## Abbreviations

AMSTAR: A MeaSurement Tool to Assess systematic Reviews.
GRADE: Grading of Recommendations Assessment, Development, and Evaluation
HS: Harmonic scalpel
RCT: Randomized control trial
SR: Systematic review
