# Supplementary material for: Performance of Harmonic devices in surgical oncology: an umbrella review of the evidence
Source: World J Surg Oncol. 2018 Jan 4;16:2. doi: 10.1186/s12957-017-1298-x (PMC5755263; doi:10.1186/s12957-017-1298-x)
Supplement: Additional file 1: Table S1. — Overview of eligible RCTs not included in systematic reviews. (DOCX 28 kb) [file 12957_2017_1298_MOESM1_ESM.docx]

**Table S1: Overview of eligible RCTs not included in systematic reviews**

| **Review (year)** | **Comparisons** | **Surgical Procedure** | **Operative time (min)** | **blood loss (mL)** | **Drainage volume (mL)** | **hospitalization (days)** | **Seroma development** | **Source of funding** | **Methodological quality** |  |
| --- | --- | --- | --- | --- | --- | --- | --- | --- | --- | --- |
| **Population:** Gastric cancer patients | | | | | | | | | | |
| Kawabata 2016 | HS vs CT | Open gastrectomy | -6.0  (-12.8 to 0.8) | 29.0  (-42.9 to 100.9) | -7.0  (-49.6 to 35.6) | -0.2  (-0.5 to 0.1) | NR | No statement of funding | High risk of bias |  |
| Oh 2016 | HS ACE vs CT | Open distal gastrectomy | -9.8  (-26.2 to 6.5) | -88.8  (-191.9 to 14.3) | 134.6  (-96.2 to 365.4) | -0.2  (-0.5 to 0.1) | NR | Industry | High risk of bias |  |
| **Population:** Breast cancer patients | | | | | | | | | | |
| Abul-Nagah 2007 | HS vs EC | Modified radical mastectomy | **20.7**  **(10.2 to 31.3)** | **- 144.2**  **(-226.1 to -62.4)** | **- 474.7**  **(-847.3 to -102.2)** | NR | 0.67  (0.21 to 2.08) | No statement of funding | Low risk of bias |  |
| Lumachi 2013 | HDD vs EC | Axillary dissection for Breast cancer | **- 13.4**  **(-21.4 to -5.4)** | **- 29.7**  **(-34.4 to -25.0)** | **- 44.3**  **(-69.9 to -18.7)** | - 0.2  (-0.5 to 0.1) | **0.47**  **(0.23 to 0.96)** | No funding | High risk of bias |  |
| Manjunath 2014 | Ultrasonic shears vs EC | Axillary dissection for Breast Cancer | NR | NR | -102.2  (-610.1 t0 405.7) | NR | NR | No statement of funding | High risk of bias |  |
| Nawaz 2015 | HS vs EC | Mastectomy and BCS with LND | NR | NR | **-142.2**  **(-165.3 to -119.2)** | NR | NR | No statement of funding | Low risk of bias |  |
| **Population:** Oral, head, and neck cancer patients | | | | | | | | | | |
| He 2011 | HS FOCUS vs CT | Neck dissection | **-47.3**  **(-57.1 to -37.5)** | NR | **-97.0**  **(-171.3 to -22.7)** | **-0.9**  **(-1.5 to -0.3)** | NR | Academic funding | Low risk of bias |  |
| **Population:** Colon cancer patients | | | | | | | | | | |
| Wilhelm 2011 | HS Wave vs CT | Left hemicolectomy | -19  (-43.6 to 5.6) | NR | NR | NR | NR | Industry | Low risk of bias |  |
| Sista 2013 | HS FOCUS vs CT | Open right hemicolectomy | **-28.0**  **(-34.5 to -21.5)** | NR | **Day 1: -30 (-17.7 to -42.3)**  **Day 2: -40 (-31.2 to 48.8)**  **Day 3: -30 (-21.4 to 38.6)** | **-3.0**  **(-1.0 to -5.0)** | NR | No statement of funding | Low risk of bias |  |

**HDD**: Harmonic dissection devices; **HS**: Harmonic scalpel; **CT**: conventional technique; **EC**: electrosurgery; **BCS**: breast-conserving surgery; **NR**: not reported.
